# Machine Learning Model Construction and Testing: Anticipating Cancer Incidence and Mortality

**DOI:** 10.3390/diseases12070139

**Published:** 2024-06-30

**Authors:** Yuanzhao Ding

**Affiliations:** School of Geography and the Environment, University of Oxford, South Parks Road, Oxford OX1 3QY, UK; armstrongding@163.com

**Keywords:** cancer, incidence, mortality, artificial intelligence, machine learning, neural network

## Abstract

In recent years, the escalating environmental challenges have contributed to a rising incidence of cancer. The precise anticipation of cancer incidence and mortality rates has emerged as a pivotal focus in scientific inquiry, exerting a profound impact on the formulation of public health policies. This investigation adopts a pioneering machine learning framework to address this critical issue, utilizing a dataset encompassing 72,591 comprehensive records that include essential variables such as age, case count, population size, race, gender, site, and year of diagnosis. Diverse machine learning algorithms, including decision trees, random forests, logistic regression, support vector machines, and neural networks, were employed in this study. The ensuing analysis revealed testing accuracies of 62.17%, 61.92%, 54.53%, 55.72%, and 62.30% for the respective models. This state-of-the-art model not only enhances our understanding of cancer dynamics but also equips researchers and policymakers with the capability of making meticulous projections concerning forthcoming cancer incidence and mortality rates. Considering sustainability, the application of this advanced machine learning framework emphasizes the importance of judiciously utilizing extensive and intricate databases. By doing so, it facilitates a more sustainable approach to healthcare planning, allowing for informed decision-making that takes into account the long-term ecological and societal impacts of cancer-related policies. This integrative perspective underscores the broader commitment to sustainable practices in both health research and public policy formulation.

## 1. Introduction

Cancer poses a formidable threat to global health and well-being [1], with a staggering estimated 18.1 million new cases and 9.6 million cancer-related deaths occurring worldwide annually [2,3]. Gender disparities are evident, with higher cancer incidence and mortality rates among males compared to females. Approximately 20% of males and 17% of females will experience cancer during their lifetime, while 13% of males and 9% of females will succumb to the disease [2,3]. Accurately predicting cancer incidence and mortality rates is a crucial pursuit in cancer research. Numerous factors, including demographic, lifestyle, environmental, and genetic elements, influence incidence rates. Moreover, healthcare accessibility and quality significantly impact mortality rates [4]. Precise predictions in these areas are vital, empowering policymakers and healthcare providers to design targeted and effective strategies and interventions, thereby combating cancer’s devastating impact on individuals and communities [5].

Traditionally, cancer prediction relied on mathematical calculations [6,7]. This process involved data collection, followed by the development of formulas connecting cancer occurrence with factors like family history, age, height, BMI, and age at first childbirth. Subsequently, these formulas were utilized for cancer prediction, and their accuracy was assessed [6]. However, such predictions heavily depended on the accuracy of the formula, which, in turn, relied on researchers’ assumptions and expertise, potentially introducing biases. To address these concerns and minimize the risk of erroneous assumptions, a novel approach has emerged—machine learning (ML). ML models establish direct connections between input data and cancer prediction outputs, bypassing the need for explicit formulas. The accuracy of ML predictions hinges on the quality and quantity of data, making it a promising technique for anticipating cancer incidence and mortality rates (Table 1).

**Figure 1:**
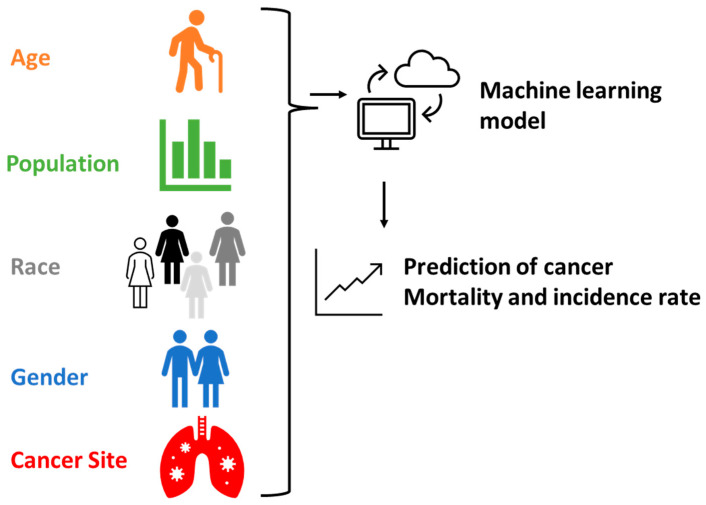
Schematic illustration showing the process of ML model setup and prediction.

The novelty of this study lies in investigating the feasibility of utilizing ML models to predict cancer incidence and mortality rates (Figure 1). The specific objectives are as follows: (1) collecting large datasets of cancer and conducting data cleaning; (2) constructing ML models using the collected data; (3) predicting cancer outcomes using the ML models; and (4) validating the prediction results and calculating accuracy. The significance of this study is in demonstrating the viability of ML models, which can then be used by policymakers to forecast cancer outbreaks and proactively plan for medical resource allocation.

## 2. Materials and Methods

### 2.1. Data Source and Selection

This study utilized data from the Centers for Disease Control and Prevention (CDC), United States Cancer Statistics (USCS), unless otherwise specified (https://www.cdc.gov/cancer/uscs/dataviz/download_data.htm, accessed on 1 March 2023). Prior to analysis, all incomplete records were removed from the dataset. Specifically, we extracted the following categories: “age”, “count”, “population”, “race”, “gender”, “site”, “year”, “incidence rate”, and “mortality rate.” To facilitate analysis, a relationship matrix was generated to compare the different categories [8,9] (Tables S1 and S2). To ensure consistency in data formatting, all data were converted to positive integers based on the classification presented in Table 2.

### 2.2. Training and Testing: Division of Data for Model Evaluation

**Figure 2:**
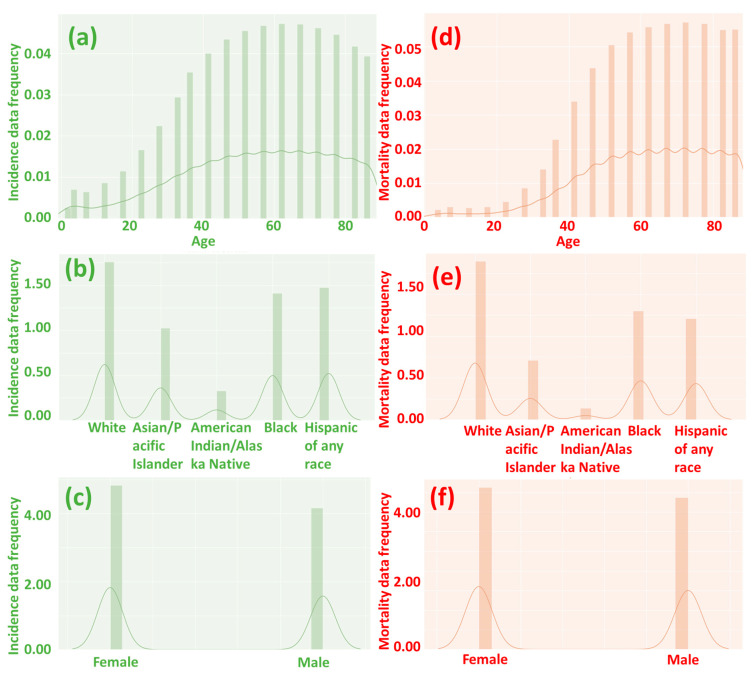
The frequency of incidence (**a**–**c**) and mortality (**d**–**f**) data by age, race, and gender. A value of 0 suggests no incidence or mortality, while a higher number indicates a higher incidence and mortality rate. When people are over 40 years old, the likelihood of cancer incidence and mortality significantly increases.

In this study, we built upon previous research [8,9] by making slight modifications to the ML models used. A total of 72,591 records (44,220 records for incidence and 28,371 records for mortality; the global distribution and data structure are shown in Figure 2) were utilized for the ML calculations, with 75% of the records used for training the models and the remaining 25% for testing. The ML models were implemented on the Anaconda 3 and Jupyter 6.3.0 platform using programming tools such as Scikit-learn, Graphviz, Numpy, Pandas, Matplotlib, and SciPy, as detailed in Tables S1 and S2. We employed five different ML methods for the calculations: decision tree, random forest, logistic regression, support vector machine (SVC), and neural network. For the random forest model, we utilized a hyper-tuning process via the random search method (Tables S3 and S4). Our neural network model comprised a total of 100 hidden layers, with each layer containing 100 nodes.

After training the models, we analyzed the testing accuracy to identify the ML model with the highest performance, referencing methodologies established in previous studies [14,15]. Subsequently, we performed a detailed investigation into two significant factors—“age” and “site”—and their influence on the incidence and mortality rates of cancer. Our analysis yielded important insights into the factors that contribute to the incidence and mortality rates of different types of cancer. Ultimately, based on our study’s results, we provided recommendations to enhance the ML models and control the most lethal cancers identified. These recommendations could assist policymakers and healthcare providers in developing targeted and effective policies and interventions to combat cancer’s pervasive and devastating impact on individuals and communities. Overall, this study underscores the potential of ML models in aiding cancer prediction and highlights the importance of continued research in this field.

## 3. Results

### 3.1. Influential Factors in Cancer Incidence

**Figure 3:**
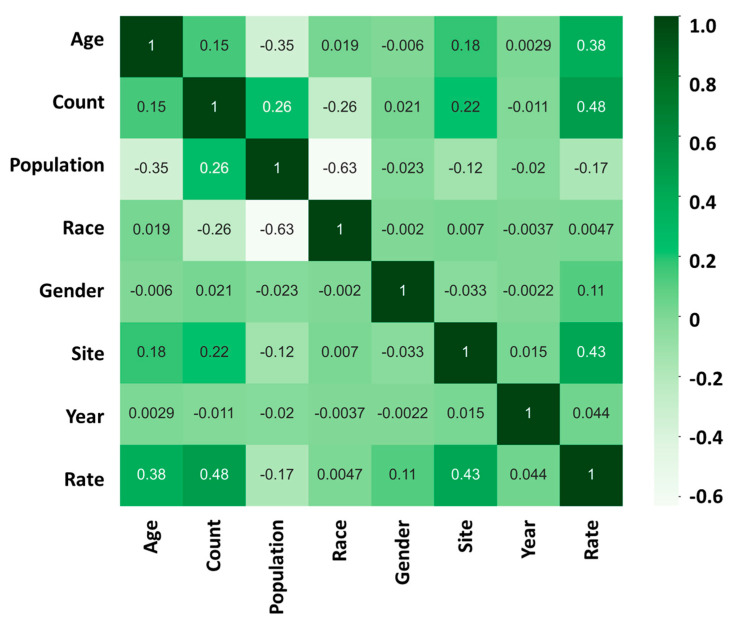
Incidence heatmap analysis showing the relationship between “age”, “count”, “population”, “race”, “gender”, “site”, “year”, and “rate” in cancer incidence rate; 1 (dark green) represents a strong positive correlation between the two sets of data; 0 represents no correlation between the two sets of data; −1 (light white) represents a strong negative correlation between the two sets of data.

After conducting a comprehensive analysis of the data, our findings revealed that the risk of developing cancer is highly influenced by two major factors: “age” and “site.” We used a heatmap analysis to evaluate the relationship between these factors and the cancer incidence rate. Our results indicate that the correlation coefficient between “age” and the cancer incidence rate was found to be +0.38 (Figure 3). This suggests that, as individuals age, they become more susceptible to developing cancer.

Furthermore, our analysis also showed that the “site” of cancer is another crucial factor that affects the incidence rate of cancer. The correlation coefficient between “site” and the cancer incidence rate was found to be +0.43 (Figure 3). This implies that the location or the specific organ where the cancer is found has a significant impact on its progression.

In contrast, our study found that factors such as “race”, “gender”, and “year of discovery” have a relatively low impact on the incidence rate of cancer. The correlation coefficient between race and the cancer incidence rate was found to be +0.0047, while that between gender and the cancer incidence rate was found to be +0.11. Similarly, the correlation coefficient between the year of discovery and the cancer incidence rate was found to be +0.044. Although these factors are important, their influence on the incidence rate of cancer is comparatively low.

Our study underscores the importance of identifying and understanding the factors that affect the incidence rate of cancer. By recognizing the significant impact of “age” and “site”, health professionals and policymakers can develop more targeted prevention and treatment strategies. Our findings provide a foundation for further research to identify additional factors that may influence the incidence rate of cancer, ultimately leading to more effective prevention and treatment methods.

**Figure 4:**
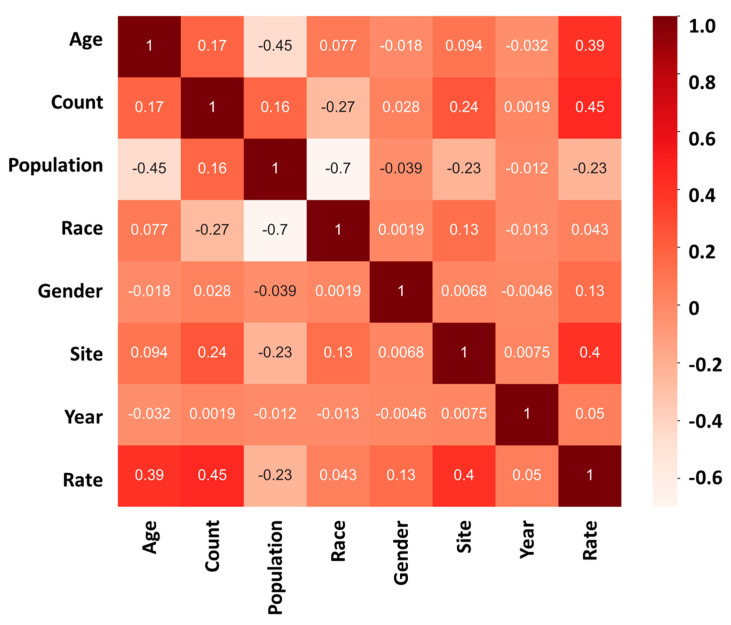
Mortality heatmap analysis showing the relationship between “age”, “count”, “population”, “race”, “gender”, “site”, “year”, and “rate” in cancer mortality rate; 1 (dark red) represents a strong positive correlation between the two sets of data; 0 represents no correlation between the two sets of data; −1 (light white) represents a strong negative correlation between the two sets of data.

The mortality rate analysis of the study further confirms the significance of age and site in influencing the outcome of cancer. The heatmap analysis revealed that “age” and “site” have a high positive correlation coefficient with the mortality rate, indicating that they are important predictors of cancer death. The correlation coefficient for “age” was found to be +0.39 (Figure 4), implying that the likelihood of cancer death increases with age. Similarly, the correlation coefficient for “site” was +0.40 (Figure 4), indicating that the location of cancer significantly affects its progression and outcome.

On the other hand, the study found that the impact of race, gender, and year of discovery on the cancer mortality rate is relatively low, with correlation coefficients of +0.043, +0.13, and +0.05, respectively. These findings suggest that, although these factors may have some influence on cancer mortality, their effect is much weaker compared to age and site.

The results of the mortality rate analysis highlight age and site as critical predictors of cancer outcomes. Older age is associated with higher cancer mortality rates, emphasizing the need for targeted prevention and treatment strategies for older populations. These findings suggest that healthcare professionals should prioritize screening and early detection programs for individuals at higher risk of developing cancer due to their age or the cancer site. Furthermore, this study underscores the necessity of additional research to elucidate the mechanisms linking age and site to cancer development and progression. Such research could inform the creation of more personalized and effective treatment approaches.

### 3.2. Advancements in ML Methods and Prediction Accuracy

In this research endeavor, we employed a diverse set of five machine learning methodologies, specifically, decision trees, random forests, logistic regression, support vector machines (SVC), and neural networks, to undertake a comprehensive evaluation and comparative analysis of their efficacy in the prediction of cancer incidence and mortality rates, as illustrated in Table 3. Subsequent to the model training phase, we conducted a rigorous performance assessment by scrutinizing the testing accuracy. The findings unequivocally demonstrated that the neural network model emerged as the most proficient, attaining an accuracy rate of 58.92% for the prediction of cancer incidence and 62.30% for mortality rate prognostication, thereby outperforming the other four models. Additionally, the neural network model exhibited superior precision levels, achieving 58.21% for cancer incidence prediction and 59.32% for mortality rate prediction, thereby surpassing the performance of the alternative models under investigation.

When comparing the neural network with the other models, namely, the decision tree, random forest, logistic regression, and SVC models, they exhibited lower testing accuracy, indicating that they are less effective in predicting cancer incidence and mortality rates. Table 3 provides a summary of the testing accuracy of each model. It is worth noting that the selection of ML methods should consider various factors, such as data characteristics, model complexity, and computational resources. Therefore, future studies may need to further evaluate and compare different ML methods with larger datasets and more complex models to improve the prediction accuracy of cancer incidence and mortality rates.

## 4. Discussion

### 4.1. Consistent Findings: Aligning with Previous Studies on Aging and Cancer Rates

Our analysis of the heatmap (Figure 3 and Figure 4) has provided valuable insights into the impact of age on the incidence and mortality rates of cancer, surpassing the influence of race, gender, and year of discovery. Notably, the majority of cancer cases occurred after the age of 40, with the highest incidence rate frequency observed in the “60–64” age category and the highest mortality rate frequency in the “70–74” age category (Figure 2a,d). These results align with previous studies that have consistently demonstrated an increasing trend in cancer rates with advancing age [16]. For instance, breast cancer incidence is typically low before the age of 30 but steadily rises, reaching its peak around the age of 80 [17]. Moreover, most patients diagnosed with invasive cancer are over the age of 65, potentially attributed to the accumulation of protein-altering mutations with age [18].

The substantial impact of age on cancer incidence and mortality rates does not come as a surprise, considering that aging is a multifaceted process that affects all systems within the body. As individuals age, cells accumulate genetic mutations and undergo various alterations that heighten the risk of cancer development [19,20]. Additionally, the aging immune system experiences a decline in efficiency, rendering it less capable of detecting and combating cancer cells [21,22]. Collectively, these factors contribute to the elevated incidence and mortality rates of cancer in older adults.

Our research outcomes underscore the paramount importance of age as a prominent risk factor for cancer, while indicating that gender and race play comparatively less significant roles in this regard. These findings highlight the imperative need for tailored strategies in cancer prevention and treatment that take into account the age of the patient. Furthermore, our results accentuate the value of ongoing investigations into the intricate interplay between the process of aging and the development of cancer. Such endeavors aim to elucidate additional intricacies within the relationship between these factors. These insights, when gained, hold the potential to substantially advance our comprehension of cancer biology and contribute to the formulation of more efficacious interventions and therapeutic approaches, particularly tailored for the aging demographic. In terms of public policy, directing limited healthcare resources more towards the elderly population (age > 40, see Figure 2) and specific cancer sites would likely contribute to enhancing the efficiency of public resource utilization and improving both life expectancy and quality of life per capita.

### 4.2. Revealing Menacing Cancer Mortality Rates

**Figure 5:**
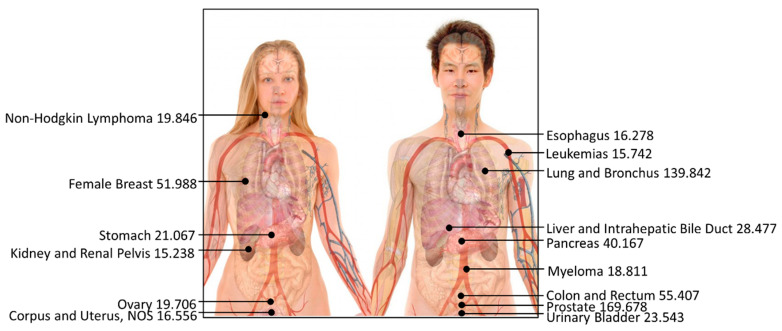
Top-15 most dangerous cancers affecting human beings (reported death rate per 100,000; the higher the data, the greater the risk of cancer). The photo has been revised from a public source authored by Mikael Häggström with Creative Commons license, and the data are presented by this study.

Our analysis of cancer mortality rates unveiled prostate cancer and lung and bronchus cancer as the most menacing, with 170 and 140 reported deaths per 100,000 individuals, respectively (Figure 5). This highlights the elevated mortality rates associated with these cancers compared to other types of cancer, as depicted in Table 4. Conversely, the incidence of testis cancer, Hodgkin lymphoma cancer, and thyroid cancer yielded the lowest reported deaths per 100,000 individuals, with rates of 0.416, 1.052, and 2.675, respectively.

It is important to note that our findings align with previous studies that have also identified prostate and lung cancer as the leading causes of cancer deaths worldwide. For instance, the World Health Organization reported that lung cancer is the most common cancer globally, accounting for 2.1 million new cases in 2018, representing 11.6% of all new cancer cases, and 1.8 million deaths, contributing to 18.4% of all cancer-related deaths [23]. Similarly, the American Cancer Society estimates that prostate cancer ranks as the second-most common cancer and the second leading cause of cancer death among men in the United States, with an estimated 248,530 new cases and 34,130 deaths in 2021 [24,25].

Given these corroborating findings, it becomes crucial to prioritize measures focused on prevention, early detection, and effective treatment for these types of cancer to alleviate the associated morbidity and mortality rates. This may involve the implementation of regular screening programs, advocating lifestyle modifications, such as smoking cessation and physical activity, and spearheading research to develop innovative therapies for advanced-stage cancers.

Our comprehensive analysis of cancer mortality rates underscores the profound significance of cancer type in predicting cancer-related outcomes. While prostate and lung cancer are associated with the highest mortality rates, testis cancer, Hodgkin lymphoma cancer, and thyroid cancer have comparatively fewer reported deaths. Recognizing these distinct patterns allows us to better comprehend the underlying risk factors and enables us to devise tailored and effective strategies to improve cancer prevention and treatment outcomes. By embracing a comprehensive approach, combining research, public health initiatives, and individualized patient care, we can strive towards mitigating the burden of cancer and enhancing the overall quality of life for those affected.

According to recent research, emerging evidence suggests that bacteria and biofilm infection may contribute to the development of prostate cancer and lung cancer [26,27,28,29]. Bacteria and biofilms are known to play a crucial role in the genesis of prostate calcifications [28,30,31], which can induce inflammation and ultimately lead to prostate cancer. Similarly, biofilm formation can lead to lung infections caused by microorganisms such as *Pseudomonas aeruginosa* and *Enterococcus faecalis* [32,33,34], potentially progressing to lung cancer.

Biofilm essentially represents a community of microorganisms that attach to a surface and embed themselves in extracellular polymeric substances, encompassing extracellular DNA, proteins, and polysaccharides [35,36,37,38,39]. In recent years, researchers have harnessed the potential of engineered biofilms for various applications, ranging from electricity generation [40,41,42] to pollutant removal [36,37,38] and concrete enhancements [43,44]. However, as biofilms also possess detrimental effects on human health, it becomes imperative to explore strategies aimed at reducing biofilm presence in the environment. For instance, researchers can investigate the development of anti-biofilm cementitious materials [45,46,47,48], offering a promising avenue to foster a cleaner environment and potentially diminish the risk of cancer. By devising effective approaches to combat biofilms, we may hold the key to lowering the incidence of prostate cancer and lung cancer, thereby enhancing overall public health.

Continuing research efforts in understanding the intricate interplay between biofilms and cancer development will pave the way for innovative interventions and preventive measures. By targeting this underlying factor, we can move closer to reducing the burden of cancer and fortifying public health on a global scale. Additionally, investigations into the mechanisms through which biofilms influence cancer initiation and progression may unveil novel therapeutic targets, opening new avenues for cancer treatment and management. Ultimately, with a multifaceted approach that encompasses both scientific advancements and public health initiatives, we can strive towards a future with improved cancer outcomes and better quality of life for affected individuals.

### 4.3. Promising Direction: ML Models for Cancer Incidence and Mortality Prediction

This paper suggests a promising direction for applying ML models to predict cancer incidence and mortality rates. Like any statistical model, prediction accuracy is influenced by several factors. In the context of cancer prediction, three key factors that affect accuracy are the number of input factors, the quantity of records used, and the choice of appropriate ML methods.

The number of input factors plays a crucial role in determining the accuracy of the predictions. This feasibility study employs seven factors (“age”, “count”, “population”, “race”, “gender”, “site”, and “year”) to predict cancer incidence and mortality rates. However, due to computational limitations, the number of input factors is currently restricted. In the future, with the availability of higher computational power, it will be possible to incorporate additional factors (e.g., height, weight, smoking habit, drinking habit, family inheritance, migration history, current city, and living environment) into the ML model. This expansion of input factors is expected to enhance the ML methods, leading to higher prediction accuracy [49].

Similarly, the quantity of records used in the study significantly impacts prediction accuracy. The current analysis employs a dataset comprising 72,591 records to calculate cancer incidence and mortality rates. As data size improves, incorporating a larger dataset (e.g., 1 million records) in the future would enable more accurate predictions of cancer incidence and mortality rates [50,51]. By harnessing a more extensive pool of data, ML models can gain a deeper understanding of underlying patterns and relationships, further contributing to improved prediction accuracy.

Furthermore, this study specifically compares different ML methods, with the neural network exhibiting the highest testing accuracy and highest precision. Nevertheless, it is worth exploring the potential of other ML methods that might yield superior results. Methods such as k-means [52], nearest neighbor [53], linear discriminant analysis [54], and hidden Markov [55] are plausible candidates for comparison to identify the most accurate method.

### 4.4. Comparative Study of Prediction Models

Several studies employ machine learning to predict cancer. For instance, one study showed how it enhances cancer diagnosis, prognosis, and personalized medicine [12], while another focuses on modeling cancer progression and treatment [56]. Additionally, a group improved breast cancer prediction accuracy using machine learning techniques [57]. Another paper evaluated five machine learning methods for early disease detection, highlighting ANNs’ superior performance [15]. Another study systematically reviewed AI techniques for COVID-19 severity assessment, highlighting XGBoost and SVM as effective in predicting severity with high sensitivity and specificity [58]. In comparison, our study’s advantage lies in using a small dataset, quickly setting up prediction models (typically within a day), and achieving reasonable testing accuracy.

## 5. Conclusions

This study highlights the transformative impact of ML models on predicting cancer incidence and mortality rates. Moving beyond traditional, assumption-laden approaches, data-driven methodologies are poised to redefine precision in cancer prediction. The ML model presented here, utilizing a robust dataset and diverse algorithms, demonstrates promising potential for enhanced accuracy and adaptability in forecasting. Achieving testing accuracies reaching up to 62.30%, this innovative approach empowers scientists and policymakers to anticipate future cancer trends and optimize healthcare strategies. Looking ahead, leveraging big data and AI holds promise for proactive interventions, facilitating timely resource allocation, and significantly improving global cancer care outcomes.

## Figures and Tables

**Table 1:** Comparison between traditional cancer and new ML models on cancer prediction.

	Breast Cancer Prediction Model	ML Model
Rationales	Use of mathematic formula to predict the cancer	Prediction of cancer via the ML algorithm
Methods	Use of data to build logic links, connecting factors (e.g., age, height, BMI) and cancer	Prediction via the “black box” without considering the logic links
Accuracy of prediction	Assumptions and connections	Quality and quantity of data
Advantages	Matured methods with clear process	Convenient and fact prediction
Limitations	Incorrect assumption and researchers’ bias	“Black swan” effect
Reference	[6,7]	[8,9,10,11,12,13]

**Table 2:** Categorizing approaches for data treatment.

**Age**	**Approximate** **Number**	**Site**	**Assigned Number**	**Year**	**Assigned Number**
≤1	1	Testis	1	2019	1
1–4	3	Hodgkin Lymphoma	2	2018	2
5–9	7	Thyroid	3	2017	3
10–14	12	Mesothelioma	4	2016	4
15–19	17	Cervix	5	2015	5
20–24	22	Brain and Other Nervous System	6	2014	6
25–29	27	Larynx	7	2013	7
30–34	32	Melanomas of the Skin	8	2012	8
35–39	37	Oral Cavity and Pharynx	9	2011	9
40–44	42	Kidney and Renal Pelvis	10	2010	10
45–49	47	Leukemias	11	2009	11
50–54	52	Esophagus	12	2008	12
55–59	57	Corpus and Uterus, NOS	13	2007	13
60–64	62	Myeloma	14	2006	14
65–69	67	Ovary	15	2005	15
70–74	72	Non-Hodgkin Lymphoma	16	2004	16
75–79	77	Stomach	17	2003	17
80–84	82	Urinary Bladder	18	2002	18
85+	87	Liver and Intrahepatic Bile Duct	19	2001	19
		Pancreas	20	2000	20
		Female Breast	21	1999	21
		Colon and Rectum	22		
		Lung and Bronchus	23		
		Prostate	24		
**Gender**	**Assigned Number**	**Race**	**Approximate Number**	**Event Type**	**Assigned Number**
Female	1	Non-Hispanic White	1	Incidence	1
Male	2	Non-Hispanic Asian/Pacific Islander	2	Mortality	2
		Non-Hispanic American Indian/Alaska Native	3		
		Non-Hispanic Black	4		
		Hispanic of any race	5		
**CIlower/CIupper**	**Approximate Number**	**Incidence/Mortality Rate**	**Assigned Number**		
[0–0.5)	0	[0–5)	0		
[0.5–1.5)	1	[5–15)	10		
[1.5–2.5)	2	[15–25)	20		
[2.5–3.5)	3	[25–35)	30		
…	…	…	…		

**Table 3:** Comparison of ML prediction method.

**Incidence rate prediction**	**Method**	**Principle**	**Testing Accuracy**	**Precision**
	Decision tree	Decision-making; nodes are features, leaves are classes	57.53%	58.02%
	Random forest	Ensemble of trees; each trained on random data subsets	57.90%	57.31%
	Logistic regression	Linear model for binary classification	50.11%	45.72%
	SVC	Finding optimal hyperplane; maximizing margin	49.99%	46.83%
	Neural network	Brain-inspired models with layered neurons	58.92%	58.21%
**Mortality rate prediction**	**Method**	**Principle**	**Testing Accuracy**	**Precision**
	Decision tree	Decision-making; nodes are features, leaves are classes	62.17%	59.18%
	Random forest	Ensemble of trees; each trained on random data subsets	61.92%	57.71%
	Logistic regression	Linear model for binary classification	54.53%	48.36%
	SVC	Finding optimal hyperplane; maximizing margin	55.72%	50.89%
	Neural network	Brain-inspired models with layered neurons	62.30%	59.32%

**Table 4:** Average mortality rate by different sites of cancer.

Sites	Reported Incidence Rate per 100,000	Reported Death Rate per 100,000
Testis	6.109	0.416
Hodgkin Lymphoma	3.357	1.052
Thyroid	15.838	2.675
Mesothelioma	5.771	5.176
Cervix	14.571	6.23
Brain and Other Nervous System	7.758	7.599
Larynx	14.946	7.642
Melanomas of the Skin	26.591	8.439
Oral Cavity and Pharynx	21.866	9.773
Kidney and Renal Pelvis	34.078	15.238
Leukemias	20.492	15.742
Esophagus	16.881	16.278
Corpus and Uterus, NOS	46.417	16.556
Myeloma	25.058	18.811
Ovary	20.202	19.706
Non-Hodgkin Lymphoma	33.855	19.849
Stomach	31.179	21.067
Urinary Bladder	58.880	23.543
Liver and Intrahepatic Bile Duct	31.211	28.477
Pancreas	40.685	40.167
Female Breast	137.875	51.988
Colon and Rectum	113.632	55.407
Lung and Bronchus	169.056	139.842
Prostate	429.041	169.678

## Data Availability

The data presented in this study is available on request from the corresponding author.

## Supplementary Materials

The following supporting information can be downloaded at: https://www.mdpi.com/article/10.3390/diseases12070139/s1, Table S1. Coding for cancer incidence prediction; Table S2. Coding for cancer incidence prediction; Table S3. Random search for random forest (incidence rate); Table S4. Random search for random forest (mortality rate).
