# Supplementary material for: Machine Learning Model Construction and Testing: Anticipating Cancer Incidence and Mortality
Source: Diseases. 2024 Jun 30;12(7):139. doi: 10.3390/diseases12070139 (PMC11275333; doi:10.3390/diseases12070139)
Supplement: Supplementary file 1 [file diseases-12-00139-s001.zip › diseases-3062881-supplementary.pdf]

Article

Machine Learning Model Construction and Testing: Anticipating Cancer Incidence and Mortality

Yuanzhao Ding
School of Geography and the Environment, University of Oxford, South Parks Road, Oxford OX1 3QY, UK; armstrongding@163.com

Table S1. Coding for cancer incidence prediction.

```
import numpy as np

import pandas as pd

import matplotlib.pyplot as plt

import seaborn as sns

import warnings

from sklearn.metrics import confusion_matrix, precision_score, recall_score, f1_score, roc_auc_score

import numpy as np

warnings.filterwarnings('ignore')



data = pd.read_csv('20230319cancerincidence.csv')

data.head(5)



sns.set(font_scale=2)



plt.hist(x = data.AGE, bins = 10, color = 'green')

plt.title('Age')

plt.xlabel('Score')

plt.ylabel('Number')

plt.show()



plt.hist(x = data.SEX, bins = 10, color = 'green')

plt.title('PercentPercentBelowBasic')

plt.xlabel('Score')
```

```
plt.ylabel('Number')
plt.show()


colors = iter(['Uncertain:red purple'])


with sns.axes_style('white'):
    g = sns.factorplot("AGE", data=data, aspect=2,
                       kind="count", color='skyblue')
g.set_xticklabels(step=5)


with sns.axes_style('white'):
    g = sns.factorplot("RACE", data=data, aspect=2,
                       kind="count", color='purple')
g.set_xticklabels(step=5)


with sns.axes_style('white'):
    g = sns.factorplot("SEX", data=data, aspect=2,
                       kind="count", color='orange')
g.set_xticklabels(step=5)


with sns.axes_style('white'):
    g = sns.factorplot("YEAR", data=data, aspect=2,
                       kind="count", color='pink')
g.set_xticklabels(step=5)


df = data.copy()
df.head()
```

```
x = data.iloc[:,0:7]

y = data.iloc[:,7]

print(x.shape)

print(y.shape)

print(x.columns)



df = data.copy()

df.head()

plt.figure(figsize = (15,12), dpi = 600)

sns.set(font_scale=1.2)

sns.heatmap(data.corr(),annot = True, cmap='Greens',annot_kws={"fontsize":20})



plt.figure(figsize=(15, 8))

sns.distplot(df.RATEROUND)

plt.ylabel('Frequency', fontsize=15)

plt.xlabel('Incidence Rate', fontsize=15)

plt.title('Incidence Rate Distribution', fontsize=15)

plt.show()



plt.figure(figsize=(15, 8))

sns.distplot(df.AGE)

plt.ylabel('Incidence Rate Frequency', fontsize=15)

plt.xlabel('Age', fontsize=15)

plt.title('Incidence Rate Distribution', fontsize=15)

plt.show()



plt.figure(figsize=(15, 8))

sns.distplot(df.RACE)

plt.ylabel('Incidence Rate Frequency', fontsize=15)

plt.xlabel('Race', fontsize=15)
```

```
plt.title('Incidence Rate Distribution', fontsize=15)
plt.show()


plt.figure(figsize=(15, 8))
sns.distplot(df.SITE)
plt.ylabel('Incidence Rate Frequency', fontsize=15)
plt.xlabel('Site', fontsize=15)
plt.title('Incidence Rate Distribution', fontsize=15)
plt.show()


plt.figure(figsize=(15, 8))
sns.distplot(df.SEX)
plt.ylabel('Incidence Rate Frequency', fontsize=15)
plt.xlabel('Sex', fontsize=15)
plt.title('Incidence Rate Distribution', fontsize=15)
plt.show()


x = pd.get_dummies(x)
x.head()


from sklearn.model_selection import train_test_split
x_train, x_test, y_train, y_test = train_test_split(x, y, test_size = 0.25, shuffle=False)
print(x_train.shape)
print(y_train.shape)
print(x_test.shape)
print(y_test.shape)
print(y_test)


from sklearn.preprocessing import StandardScaler
```

```
sc = StandardScaler()
x_train = sc.fit_transform(x_train)
x_test = sc.fit_transform(x_test)
x_train = pd.DataFrame(x_train)
x_train.head()
print(x_train)
```

```
from sklearn.tree import DecisionTreeClassifier
from sklearn.metrics import confusion_matrix
model = DecisionTreeClassifier()
model.fit(x_train, y_train)
y_pred = model.predict(x_test)
print("Training Accuracy :", model.score(x_train, y_train))
print("Testing Accuaracy :", model.score(x_test, y_test))
precision = precision_score(y_test, y_pred, average='weighted')
recall = recall_score(y_test, y_pred, average='weighted')
f1 = f1_score(y_test, y_pred, average='weighted')
print("Precision:", precision)
print("Recall:", recall)
print("F1-Score:", f1)
cm = confusion_matrix(y_test, y_pred)
np.set_printoptions(threshold=50000)
pd.set_option('max_colwidth',1)
print(cm)
print(y_pred)
```

```
from sklearn.ensemble import RandomForestClassifier
model = RandomForestClassifier()
model.fit(x_train, y_train)
y_pred = model.predict(x_test)
print("Training Accuracy :", model.score(x_train, y_train))
print("Testing Accuracy :", model.score(x_test, y_test))
```

```
precision = precision_score(y_test, y_pred, average='weighted')
recall = recall_score(y_test, y_pred, average='weighted')
f1 = f1_score(y_test, y_pred, average='weighted')
print("Precision:", precision)
print("Recall:", recall)
print("F1-Score:", f1)
cm = confusion_matrix(y_test, y_pred)
np.set_printoptions(threshold=50000)
pd.set_option('max_colwidth',1)
print(cm)
print(y_pred)
```

```
from sklearn.model_selection import cross_val_score
cvs = cross_val_score(estimator = model, X = x_train, y = y_train, cv = 10)
precision = precision_score(y_test, y_pred, average='weighted')
recall = recall_score(y_test, y_pred, average='weighted')
f1 = f1_score(y_test, y_pred, average='weighted')
print("Precision:", precision)
print("Recall:", recall)
print("F1-Score:", f1)
print(cvs)
print(y_pred)
print("Mean Accuracy :", cvs.mean())
print("Variance :", cvs.std())
```

```
from sklearn.linear_model import LogisticRegression
model = LogisticRegression()
model.fit(x_train, y_train)
y_pred = model.predict(x_test)
print("Training Accuracy :", model.score(x_train, y_train))
print("Testing Accuracy :", model.score(x_test, y_test))
precision = precision_score(y_test, y_pred, average='weighted')
```

```
recall = recall_score(y_test, y_pred, average='weighted')
f1 = f1_score(y_test, y_pred, average='weighted')
print("Precision:", precision)
print("Recall:", recall)
print("F1-Score:", f1)
cm = confusion_matrix(y_test, y_pred)
np.set_printoptions(threshold=50000)
pd.set_option('max_colwidth',1)
print(cm)
print(y_pred)
```

```
from sklearn.svm import SVC
model = SVC()
model.fit(x_train, y_train)
y_pred = model.predict(x_test)
print("Training Accuracy :", model.score(x_train, y_train))
print("Testing Accuracy :", model.score(x_test, y_test))
precision = precision_score(y_test, y_pred, average='weighted')
recall = recall_score(y_test, y_pred, average='weighted')
f1 = f1_score(y_test, y_pred, average='weighted')
print("Precision:", precision)
print("Recall:", recall)
print("F1-Score:", f1)
cm = confusion_matrix(y_test, y_pred)
np.set_printoptions(threshold=50000)
pd.set_option('max_colwidth',1)
print(cm)
print(y_pred)
```

```
from sklearn.neural_network import MLPClassifier
model = MLPClassifier(hidden_layer_sizes = (100, 100), activation ='relu',
                      solver = 'adam', max_iter = 50)
```

```
model.fit(x_train, y_train)
y_pred = model.predict(x_test)
print("Training Accuracy :", model.score(x_train, y_train))
print("Testing Accuracy :", model.score(x_test, y_test))
precision = precision_score(y_test, y_pred, average='weighted')
recall = recall_score(y_test, y_pred, average='weighted')
f1 = f1_score(y_test, y_pred, average='weighted')
print("Precision:", precision)
print("Recall:", recall)
print("F1-Score:", f1)
cm = confusion_matrix(y_test, y_pred)
np.set_printoptions(threshold=50000, linewidth=1)
pd.set_option('max_colwidth',1)
print(cm)
print(y_pred)
```

```
df = pd.read_csv("20230319cancerincidence.csv", index_col=0)
ncol = len(df.axes[1])
nrow=len(df.axes[0])
print("%s x %s" % (nrow, ncol)) #check dimension
print (df.dtypes) #check data types
df.groupby('RATEROUND').count()
n = ncol
for i in range(0,n):
    if (df.iloc[:,i].dtype==object):
     A= df.iloc[:,i].unique()
     map_to_int = {name: n for n, name in enumerate(A)}
     df.iloc[:,i] = df.iloc[:,i].replace(map_to_int)
print (df.dtypes)
df1=df.iloc[:,0:n]
features = list(df1.columns[:(n-2)])
print("* features:", features, sep="\n")
df1.rename(columns={'y':'Target'}, inplace=True)
```

```
y = df1["RATEROUND"]
X = df1[features]


%matplotlib
import matplotlib.pyplot as plt
fig = plt.figure(figsize=(20, 20))
df1.hist(bins=20)
plt.show()
corr_df = df1.corr()


%matplotlib inline
import seaborn
import matplotlib.pyplot as plt
print(" CorrelationMatrix")
mask = np.zeros_like(corr_df)
mask[np.triu_indices_from(mask)] = True
seaborn.heatmap(corr_df, cmap='RdYlGn_r', vmax=1.0, vmin=-1 ,mask = mask,  linewidths=3,fmt='.1f')
plt.yticks(rotation=0,fontsize=10)
plt.xticks(rotation=90,fontsize=10)
plt.show()
print(__doc__)


import numpy as np
from time import time
from operator import itemgetter
from scipy.stats import randint as sp_randint
from sklearn.model_selection import GridSearchCV, RandomizedSearchCV
from sklearn.datasets import load_digits
from sklearn.ensemble import RandomForestClassifier
clf = RandomForestClassifier(n_estimators=20)
def report(grid_scores, n_top=3):
```

```
    top_scores = sorted(grid_scores, key=itemgetter(1), reverse=True)[:n_top]
    for i, score in enumerate(top_scores):
        print("Model with rank: {0}".format(i + 1))
        print("Mean validation score: {0:.3f} (std: {1:.3f})".format(
              score.mean_validation_score,
              np.std(score.cv_validation_scores)))
        print("Parameters: {0}".format(score.parameters))
        print("")
param_dist = {"max_depth": [3, None],
              "max_features": sp_randint(1, 11),
              "min_samples_split": sp_randint(1, 11),
              "min_samples_leaf": sp_randint(1, 11),
              "bootstrap": [True, False],
              "criterion": ["gini", "entropy"]}
n_iter_search = 20
random_search = RandomizedSearchCV(clf, param_distributions=param_dist,
                                   n_iter=n_iter_search)
start = time()
random_search.fit(X, y)
print("RandomizedSearchCV took %.2f seconds for %d candidates"
      " parameter settings." % ((time() - start), n_iter_search))
pd.DataFrame(random_search.cv_results_)
```

Table S2. Coding for cancer mortility prediction.

```
import numpy as np

import pandas as pd

import matplotlib.pyplot as plt

import seaborn as sns

import warnings

from sklearn.metrics import confusion_matrix, precision_score, recall_score, f1_score, roc_auc_score

import numpy as np

warnings.filterwarnings('ignore')



data = pd.read_csv('20230319cancermortility.csv')

data.head(5)



sns.set(font_scale=2)



plt.hist(x = data.AGE, bins = 10, color = 'red')

plt.title('Age')

plt.xlabel('Score')

plt.ylabel('Number')

plt.show()



plt.hist(x = data.SEX, bins = 10, color = 'red')

plt.title('PercentPercentBelowBasic')

plt.xlabel('Score')

plt.ylabel('Number')

plt.show()



colors = iter(['Uncertain:red purple'])
```

```
with sns.axes_style('white'):
    g = sns.factorplot("AGE", data=data, aspect=2,
                       kind="count", color='red')
g.set_xticklabels(step=5)
```

```
df = data.copy()
df.head()
x = data.iloc[:,0:7]
y = data.iloc[:,7]
print(x.shape)
print(y.shape)
print(x.columns)
```

```
df = data.copy()
df.head()
plt.figure(figsize = (15,12), dpi = 600)
sns.set(font_scale=1.2)
sns.heatmap(data.corr(),annot = True, cmap='Reds',annot_kws={"fontsize":20})
```

```
plt.figure(figsize=(15, 8))
sns.distplot(df.RATEROUND)
plt.ylabel('Frequency', fontsize=15)
plt.xlabel('Mortility Rate', fontsize=15)
plt.title('Mortility Rate Distribution', fontsize=15)
plt.show()
```

```
plt.figure(figsize=(15, 8))
sns.distplot(df.AGE)
plt.ylabel('Mortility Rate Frequency', fontsize=15)
```

```
plt.xlabel('Age', fontsize=15)
plt.title('Mortility Rate Distribution', fontsize=15)
plt.show()


plt.figure(figsize=(15, 8))
sns.distplot(df.RACE)
plt.ylabel('Mortality Rate Frequency', fontsize=15)
plt.xlabel('Race', fontsize=15)
plt.title('Mortality Rate Distribution', fontsize=15)
plt.show()


plt.figure(figsize=(15, 8))
sns.distplot(df.SITE)
plt.ylabel('Mortality Rate Frequency', fontsize=15)
plt.xlabel('Site', fontsize=15)
plt.title('Mortality Rate Distribution', fontsize=15)
plt.show()


plt.figure(figsize=(15, 8))
sns.distplot(df.SEX)
plt.ylabel('Mortality Rate Frequency', fontsize=15)
plt.xlabel('Sex', fontsize=15)
plt.title('Mortality Rate Distribution', fontsize=15)
plt.show()


x = pd.get_dummies(x)
x.head()


from sklearn.model_selection import train_test_split
```

```
x_train, x_test, y_train, y_test = train_test_split(x, y, test_size = 0.25, shuffle=False)
print(x_train.shape)
print(y_train.shape)
print(x_test.shape)
print(y_test.shape)
print(y_test)
```

```
from sklearn.preprocessing import StandardScaler
sc = StandardScaler()
x_train = sc.fit_transform(x_train)
x_test = sc.fit_transform(x_test)
x_train = pd.DataFrame(x_train)
x_train.head()
print(x_train)
```

```
from sklearn.tree import DecisionTreeClassifier
from sklearn.metrics import confusion_matrix
model = DecisionTreeClassifier()
model.fit(x_train, y_train)
y_pred = model.predict(x_test)
print("Training Accuracy :", model.score(x_train, y_train))
print("Testing Accuaracy :", model.score(x_test, y_test))
precision = precision_score(y_test, y_pred, average='weighted')
recall = recall_score(y_test, y_pred, average='weighted')
f1 = f1_score(y_test, y_pred, average='weighted')
print("Precision:", precision)
print("Recall:", recall)
print("F1-Score:", f1)
cm = confusion_matrix(y_test, y_pred)
np.set_printoptions(threshold=50000)
pd.set_option('max_colwidth',1)
print(cm)
```

```
print(y_pred)


from sklearn.ensemble import RandomForestClassifier
model = RandomForestClassifier()
model.fit(x_train, y_train)
y_pred = model.predict(x_test)
print("Training Accuracy :", model.score(x_train, y_train))
print("Testing Accuracy :", model.score(x_test, y_test))
precision = precision_score(y_test, y_pred, average='weighted')
recall = recall_score(y_test, y_pred, average='weighted')
f1 = f1_score(y_test, y_pred, average='weighted')
print("Precision:", precision)
print("Recall:", recall)
print("F1-Score:", f1)
cm = confusion_matrix(y_test, y_pred)
np.set_printoptions(threshold=50000)
pd.set_option('max_colwidth',1)
print(cm)
print(y_pred)


from sklearn.model_selection import cross_val_score
cvs = cross_val_score(estimator = model, X = x_train, y = y_train, cv = 10)
print(cvs)
print(y_pred)
precision = precision_score(y_test, y_pred, average='weighted')
recall = recall_score(y_test, y_pred, average='weighted')
f1 = f1_score(y_test, y_pred, average='weighted')
print("Precision:", precision)
print("Recall:", recall)
print("F1-Score:", f1)
print("Mean Accuracy :", cvs.mean())
print("Variance :", cvs.std())
```

```
from sklearn.linear_model import LogisticRegression
model = LogisticRegression()
model.fit(x_train, y_train)
y_pred = model.predict(x_test)
print("Training Accuracy :", model.score(x_train, y_train))
print("Testing Accuracy :", model.score(x_test, y_test))
precision = precision_score(y_test, y_pred, average='weighted')
recall = recall_score(y_test, y_pred, average='weighted')
f1 = f1_score(y_test, y_pred, average='weighted')
print("Precision:", precision)
print("Recall:", recall)
print("F1-Score:", f1)
cm = confusion_matrix(y_test, y_pred)
np.set_printoptions(threshold=50000)
pd.set_option('max_colwidth',1)
print(cm)
print(y_pred)
```

```
from sklearn.svm import SVC
model = SVC()
model.fit(x_train, y_train)
y_pred = model.predict(x_test)
print("Training Accuracy :", model.score(x_train, y_train))
print("Testing Accuracy :", model.score(x_test, y_test))
precision = precision_score(y_test, y_pred, average='weighted')
recall = recall_score(y_test, y_pred, average='weighted')
f1 = f1_score(y_test, y_pred, average='weighted')
print("Precision:", precision)
print("Recall:", recall)
print("F1-Score:", f1)
cm = confusion_matrix(y_test, y_pred)
```

```
np.set_printoptions(threshold=50000)
pd.set_option('max_colwidth',1)
print(cm)
print(y_pred)


from sklearn.neural_network import MLPClassifier
model = MLPClassifier(hidden_layer_sizes = (100, 100), activation ='relu',
          solver = 'adam', max_iter = 50)
model.fit(x_train, y_train)
y_pred = model.predict(x_test)
print("Training Accuracy :", model.score(x_train, y_train))
print("Testing Accuracy :", model.score(x_test, y_test))
precision = precision_score(y_test, y_pred, average='weighted')
recall = recall_score(y_test, y_pred, average='weighted')
f1 = f1_score(y_test, y_pred, average='weighted')
print("Precision:", precision)
print("Recall:", recall)
print("F1-Score:", f1)
cm = confusion_matrix(y_test, y_pred)
np.set_printoptions(threshold=50000, linewidth=1)
pd.set_option('max_colwidth',1)
print(cm)
print(y_pred)


df = pd.read_csv("20230319cancermortility.csv", index_col=0)
ncol = len(df.axes[1])
nrow=len(df.axes[0])
print("%s x %s" % (nrow, ncol)) #check dimension
print (df.dtypes) #check data types
df.groupby('RATEROUND').count()
n = ncol
for i in range(0,n):
```

```
    if (df.iloc[:,i].dtype==object):
     A= df.iloc[:,i].unique()
     map_to_int = {name: n for n, name in enumerate(A)}
     df.iloc[:,i] = df.iloc[:,i].replace(map_to_int)
print (df.dtypes)
df1=df.iloc[:,0:n]
features = list(df1.columns[:(n-2)])
print("* features:", features, sep="\n")
df1.rename(columns={'y':'Target'}, inplace=True)
y = df1["RATEROUND"]
X = df1[features]


%matplotlib
import matplotlib.pyplot as plt
fig = plt.figure(figsize=(20, 20))
df1.hist(bins=20)
plt.show()
corr_df = df1.corr()


%matplotlib inline
import seaborn
import matplotlib.pyplot as plt
print(" CorrelationMatrix")
mask = np.zeros_like(corr_df)
mask[np.triu_indices_from(mask)] = True
seaborn.heatmap(corr_df, cmap='RdYlGn_r', vmax=1.0, vmin=-1 ,mask = mask,  linewidths=3,fmt='.1f')
plt.yticks(rotation=0,fontsize=10)
plt.xticks(rotation=90,fontsize=10)
plt.show()
print(__doc__)
```

```
import numpy as np
from time import time
from operator import itemgetter
from scipy.stats import randint as sp_randint
from sklearn.model_selection import GridSearchCV, RandomizedSearchCV
from sklearn.datasets import load_digits
from sklearn.ensemble import RandomForestClassifier
clf = RandomForestClassifier(n_estimators=20)
def report(grid_scores, n_top=3):
    top_scores = sorted(grid_scores, key=itemgetter(1), reverse=True)[:n_top]
    for i, score in enumerate(top_scores):
        print("Model with rank: {0}".format(i + 1))
        print("Mean validation score: {0:.3f} (std: {1:.3f})".format(
              score.mean_validation_score,
              np.std(score.cv_validation_scores)))
        print("Parameters: {0}".format(score.parameters))
        print("")
param_dist = {"max_depth": [3, None],
              "max_features": sp_randint(1, 11),
              "min_samples_split": sp_randint(1, 11),
              "min_samples_leaf": sp_randint(1, 11),
              "bootstrap": [True, False],
              "criterion": ["gini", "entropy"]}
n_iter_search = 20
random_search = RandomizedSearchCV(clf, param_distributions=param_dist,
                                   n_iter=n_iter_search)
start = time()
random_search.fit(X, y)
print("RandomizedSearchCV took %.2f seconds for %d candidates"
      " parameter settings." % ((time() - start), n_iter_search))
pd.DataFrame(random_search.cv_results_)
```

Table S3. Random search for random forest (incidence rate).

rank_test_score	mean_test_score	param_bootstrap	param_criterion	param_max_depth	param_max_features	param_min_samples_leaf	param_min_samples_split
1	0.848598	True	gini	None	5	7	2
2	0.847897	True	entropy	None	5	7	10
3	0.842017	True	gini	None	3	4	3
4	0.840660	False	gini	None	3	6	6
5	0.427748	False	gini	3	2	10	6
6	0.382632	False	gini	3	1	10	2
7	0.370059	False	entropy	3	5	8	2
8	0.359000	True	gini	3	1	1	7
9	0.325554	True	entropy	3	1	5	4
10	NaN	True	gini	None	9	2	1
11	NaN	True	entropy	3	10	2	7
12	NaN	False	gini	3	6	5	9
13	NaN	True	entropy	3	10	2	7
14	NaN	False	entropy	None	8	6	9
15	NaN	False	entropy	None	10	4	9
16	NaN	False	entropy	3	9	2	6
17	NaN	False	entropy	None	8	10	1
18	NaN	True	entropy	3	10	9	10
19	NaN	True	entropy	None	7	4	6
20	NaN	False	gini	3	10	10	9

Table S4. Random search for random forest (mortality rate).

rank_test_score	mean_test_score	param_bootstrap	param_criterion	param_max_depth	param_max_features	param_min_samples_leaf	param_min_samples_split
1	0.879490	False	entropy	None	4	5	5
2	0.873357	True	entropy	None	5	7	5
3	0.859751	True	entropy	None	4	10	6
4	0.836136	False	entropy	None	2	6	3
5	0.493426	False	entropy	3	5	8	10
6	0.487435	True	gini	3	3	7	6
7	0.426492	True	entropy	3	1	3	5
8	0.417081	False	entropy	3	1	5	4
9	NaN	False	entropy	None	9	2	6
10	NaN	True	gini	3	7	6	7
11	NaN	True	entropy	3	9	10	6
12	NaN	False	entropy	None	10	6	9
13	NaN	True	gini	3	8	4	8
14	NaN	True	gini	3	8	1	3
15	NaN	True	entropy	None	7	10	4
16	NaN	False	entropy	3	9	1	3
17	NaN	True	gini	None	7	10	9
18	NaN	False	entropy	None	4	10	1
19	NaN	False	gini	None	9	5	9
20	NaN	True	entropy	None	7	9	4
